# Supplementary material for: Analysis of Dietary Patterns Associated with Kidney Stone Disease Based on Data-Driven Approaches: A Case-Control Study in Shanghai
Source: Nutrients. 2024 Jan 9;16(2):214. doi: 10.3390/nu16020214 (PMC10818537; doi:10.3390/nu16020214)
Supplement: Supplementary file 1 [file nutrients-16-00214-s001.zip › nutrients-2793119-supplementary.pdf]

Analysis of dietary patterns associated with kidney stone disease based on data-driven approaches

Yifei Wang[1, †], Shaojie Liu[2, †], Qi Zhao[3,4], Na Wang[3,4], Xing Liu[3,4], Tiejun Zhang[3,4], Gengsheng He[1,3], Genming Zhao[3,4], Yonggen Jiang[5] and Bo Chen[1, 3, *]

[1] Department of Nutrition and food hygiene, School of Public Health, Fudan University, Shanghai 200032, China; wangyf22@m.fudan.edu.cn (Y.W.); gshe@shmu.edu.cn (G.H.)

[2] Department of Clinical Nutrition, The First Affiliated Hospital of Xiamen University, School of Medicine, Xiamen University, Xiamen 361003, China; liushaojie@fudan.edu.cn (S.L.)

[3] Key Lab of Public Health Safety of the Ministry of Education, School of Public Health, Fudan University, Shanghai 200032, China; zhaoqi@shmu.edu.cn (Q.Z.); na.wang@fudan.edu.cn (N.W.); liuxing@fudan.edu.cn (X.L.); tjzhang@shmu.edu.cn (T.Z.); gmzhao@shmu.edu.cn (G.Z.)

[4] Department of Epidemiology, School of Public Health, Fudan University, Shanghai 200032, China

[5] Songjiang District Center for Disease Control and Prevention, Shanghai 201620, China; sjjktj@hotmail.com

[†] These authors contributed equally to this work.

* Authors to whom correspondence should be addressed. Email: chenb@fudan.edu.cn

Supplements

Supplementary table S1. Characteristics of all subjects

Covariables	Male			Female			Total		
	Cases (n = 465)	None-cases (n = 18414)	*P*	Cases (n = 601)	None-cases (n = 27178)	*P*	Cases (n = 1066)	None-cases (n = 45592)	*P*
Age	48.1±10.0	58.2±11.0	<0.001	47.2±9.4	56.5±11.0	<0.001	47.6±9.79.7	57.2±11.0	<0.001

BMI	25.0±3.4	24.6±3.2	0.006	23.2±3.3	24.1±3.4	<0.001	24.0±3.5	24.3±3.3	0.008
Education			<0.001			<0.001			<0.001
Elementary school and below	18.7%	36.1%		26.3%	47.9%		23.0%	43.1%	
Junior high school	50.8%	42.9%		47.8%	34.7%		49.1%	38.0%	
Senior high school	15.5%	14.4%		14.0%	10.8%		14.6%	12.3%	
Junior college and above	15.1%	6.6%		12.0%	6.6%		13.2%	6.6%	
Smoking			0.054			<0.001			0.711
Yes	55.1%	59.6%		1.3%	0.3%		24.8%	24.2%	
No	44.9%	40.4%		98.7%	99.7%		75.2%	75.8%	
Alcohol use			0.120			0.657			0.741
Yes	28.6%	32.1%		1.0%	0.8%		13.0%	87.0%	
No	71.4%	67.9%		99.0%	99.2%		13.4%	85.6%	
Water intake			0.447			0.413			0.405
< 500 ml	1.3%	1.2%		5.8%	5.1%		3.8%	3.5%	
500 ~ 1000 ml	6.9%	6.9%		17.8%	17.8%		13.0%	13.4%	
1000 ~ 1500 ml	22.4%	24.2%		33.6%	33.5%		28.7%	29.7%	
1500 ~ 2000 ml	30.1%	28.3%		23.0%	25.1%		26.1%	26.4%	
2000 ~ 3000 ml	23.0%	25.2%		12.6%	12.9%		17.2%	17.9%	
3000 ~ 4000 ml	9.7%	9.5%		6.3%	4.6%		7.8%	6.6%	
> 4000 ml	6.7%	4.7%		0.8%	1.1%		3.4%	2.6%	

Tea drinking			0.645			0.023			0.061
Yes	57.4%	58.6%		13.1%	10.2%		67.5%	32.5%	
No	42.6%	41.4%		86.9%	89.8%		70.2%	29.8%	
Urinary pH	5.93±0.67	5.91±0.66	0.562	6.03±0.77	5.93±0.74	<0.001	5.99±0.73	5.92±0.71	0.003

Supplementary table S2. Dietary patterns yielded by PCA

	PC1	PC2	PC3	PC4	PC5	PC6	PC7	PC8	PC9	PC10
Male										
Rice	0.01	0.08	-0.02	0.07	-0.05	0.17	-0.09	**0.69**	**-0.56**	0.12
Wheat	0.14	-0.16	-0.05	-0.11	0.13	0.18	-0.03	**0.39**	**0.41**	**0.34**
Whole grain and mixed beans	0.15	-0.15	-0.18	-0.3	0.16	-0.07	-0.22	-0.13	-0.20	**0.32**
Potatoes	0.16	-0.08	-0.22	-0.2	0.18	0.15	-0.23	-0.22	-0.19	0.13
Fresh vegetables	0.14	0.03	**-0.44**	0.15	-0.29	-0.27	0.07	0.15	0	-0.09
Dark vegetables	0.16	0.03	**-0.45**	0.10	-0.18	**-0.34**	0.11	0.15	0.05	0.01
Mushrooms	0.24	-0.01	-0.22	-0.03	-0.08	0.11	0.12	-0.18	0.02	0.02
Fruits	0.16	-0.17	-0.14	**-0.31**	-0.11	-0.24	0.09	-0.06	0.04	0.09
Dairy	0.09	-0.15	0.04	**-0.37**	-0.15	0	0.25	-0.01	-0.08	-0.23
Yogurt	0.19	0.10	0.29	**-0.33**	-0.20	-0.14	0.02	0.08	-0.06	0.05
Pork	0.26	0.14	-0.01	0.19	0.02	-0.21	-0.05	-0.02	0.10	0.16
Meat from other livestock meat	**0.32**	0.18	0.28	0.02	-0.10	-0.10	0.05	-0.01	0.02	0.14
Poultry	**0.32**	0.18	0.22	0.04	-0.10	-0.02	0	-0.04	0.04	0.09
Innards	0.17	-0.02	0.14	0.27	0.12	-0.16	0.25	-0.06	-0.09	0.10
Freshwater fish	0.24	0.22	-0.07	0.06	-0.11	0.25	-0.14	0	0.06	-0.24
Marine fish	0.23	0.11	-0.16	0.05	-0.06	**0.37**	-0.04	-0.03	0	**-0.30**

Crustacean	0.29	0.29	0.25	-0.07	-0.10	-0.01	-0.13	0.02	-0.04	-0.07
Soymilk	0.12	-0.26	0.04	-0.03	-0.17	**0.35**	0.26	-0.04	-0.13	0.13
Tofu	0.26	-0.04	-0.12	0.08	0.05	**0.33**	0.11	-0.10	0.04	-0.01
Eggs	0.18	-0.02	-0.13	-0.03	0.12	0.06	**-0.43**	0.03	0.24	0.01
Nuts	0.14	-0.09	0.02	-0.18	**0.34**	0.01	0.11	**0.37**	0.14	-0.21
Carbonated drinks	0.07	**-0.39**	0.17	0.27	-0.20	-0.07	**-0.36**	0.07	0.07	-0.07
Pure juices	0.10	**-0.36**	0.04	-0.07	-0.26	0.03	0.12	-0.04	-0.18	-0.19
Other sugar drinks	0.08	**-0.44**	0.16	0.22	-0.20	-0.06	**-0.32**	-0.03	-0.02	-0.08
Candies and chocolates	0.16	-0.09	0.09	-0.18	0.23	-0.28	-0.13	0.02	-0.24	-0.09
Fried dough foods	0.15	-0.23	0.12	0.18	0.14	0.05	0.27	-0.02	0.15	0.28
Pickles	0.13	0.04	-0.07	0.19	**0.34**	-0.08	-0.07	-0.22	**-0.37**	-0.09
Processed meat	0.17	-0.13	-0.01	0.29	0.29	-0.03	0.25	0.06	-0.14	-0.07
Pastries	0.12	-0.12	0.07	-0.08	**0.30**	-0.15	0.04	0.11	0.20	**-0.50**

	PC11	PC12	PC13	PC14	PC15	PC16	PC17	PC18	PC19	PC20
Rice	-0.28	0.15	0.06	0	0.07	-0.04	-0.15	0.06	0.05	0.07
Wheat	0.27	0.03	0.17	-0.23	-0.01	0.05	0.07	0.04	0.29	**-0.39**
Whole grain and mixed beans	0	-0.07	0.18	0.15	0.01	-0.26	0.04	-0.15	**-0.41**	0.12
Potatoes	-0.19	-0.16	0.26	-0.24	-0.20	**0.36**	0.06	-0.07	-0.06	-0.14
Fresh vegetables	0.01	-0.09	-0.09	-0.10	-0.12	0.15	0.15	-0.04	-0.09	-0.17
Dark vegetables	0.04	-0.05	-0.06	0.03	-0.10	0.01	0.06	0.09	-0.13	0.04
Mushrooms	-0.05	-0.02	0.22	0	0.09	0.19	-0.16	0.19	0.28	**0.40**
Fruits	-0.03	0.09	0.08	**0.42**	**0.38**	-0.20	**-0.33**	0.11	0.20	-0.20
Dairy	0.04	**0.51**	0.10	-0.20	**-0.41**	-0.24	0.19	0.21	-0.04	0.07
Yogurt	0.08	0.10	-0.01	-0.11	0.11	**0.31**	-0.02	0.09	-0.16	-0.03
Pork	-0.20	0.20	-0.14	-0.04	0.04	-0.02	0.09	-0.21	0.03	0.07
Meat from other livestock meat	0.08	0	0.08	0.05	0.05	0.05	0.17	-0.10	0.01	0.06

Poultry	-0.08	0	0	0.11	-0.05	0.10	0.05	-0.03	0.11	0.08
Innards	0.04	-0.14	0.2	-0.12	**-0.46**	-0.19	**-0.57**	-0.05	0.10	-0.02
Freshwater fish	0.02	-0.13	0	0.05	-0.08	-0.10	-0.26	0.08	-0.22	-0.28
Marine fish	0.10	-0.10	0.09	-0.01	0.16	**-0.42**	0.15	0.05	0.01	-0.09
Crustacean	0.08	-0.12	0.05	-0.05	0.04	-0.08	0.02	-0.01	-0.13	-0.11
Soymilk	-0.02	0.02	-0.37	**0.45**	-0.28	0.16	0.05	**-0.30**	-0.02	-0.24
Tofu	-0.21	-0.01	-0.1	0.03	0.04	0.11	0.11	0.2	0.19	0.25
Eggs	0.05	**0.39**	-0.37	-0.11	-0.1	-0.14	-0.24	-0.28	0.04	0.23
Nuts	**0.33**	-0.27	-0.15	0.19	-0.06	0.20	-0.11	0.11	-0.25	**0.36**
Carbonated drinks	-0.01	0.05	0.2	0.17	-0.03	0.10	0.12	0.14	0.05	0.06
Pure juices	0.12	-0.15	-0.15	**-0.48**	**0.36**	0.05	-0.21	**-0.38**	0.03	0.06
Other sugar drinks	0.09	-0.06	0.05	0.05	-0.09	-0.03	-0.02	0.2	-0.1	0.01
Candies and chocolates	-0.08	**-0.38**	-0.35	-0.06	-0.12	-0.25	0.27	0.1	**0.45**	-0.07
Fried dough foods	**-0.32**	-0.03	-0.24	-0.26	0.21	-0.18	0.04	**0.34**	**-0.40**	-0.07
Pickles	**0.33**	**0.36**	-0.20	0	0.15	0.26	-0.1	**0.30**	0.01	-0.28
Processed meat	0.24	0.14	**0.32**	0.08	0.16	-0.10	**0.30**	**-0.35**	-0.03	0.05
Pastries	**-0.53**	0.10	0.15	0.08	0.04	0.17	-0.06	-0.18	-0.05	-0.22

	PC21	PC22	PC23	PC24	PC25	PC26	PC27	PC28	PC29
Rice	-0.02	-0.01	0	-0.08	-0.03	0.01	0.02	0	0.05
Wheat	0.17	0.15	0.02	-0.05	0.02	0	-0.03	-0.01	-0.03
Whole grain and mixed beans	**0.43**	0.25	0	0.09	0.05	-0.05	0.06	0.07	0
Potatoes	**-0.32**	**-0.39**	-0.04	-0.11	-0.08	0.04	-0.1	-0.01	0.02
Fresh vegetables	0.11	-0.14	0.05	0.06	0.12	-0.10	**0.60**	0.14	-0.02
Dark vegetables	-0.11	0.20	**-0.31**	0.01	-0.23	0.11	**-0.54**	-0.16	-0.02
Mushrooms	-0.15	**0.43**	**0.33**	-0.12	-0.15	-0.22	0.15	-0.04	0.02
Fruits	-0.15	**-0.34**	-0.02	-0.04	0.04	0.12	0.06	-0.04	-0.03

Dairy	0.07	-0.11	0.13	-0.08	-0.06	0.10	-0.01	-0.03	-0.01	
Yogurt	-0.27	0.19	-0.13	**0.40**	**0.38**	-0.21	-0.10	0.19	-0.02	
Pork	0.11	-0.2	**0.46**	-0.22	**0.39**	-0.19	**-0.34**	-0.08	-0.05	
Meat from other livestock meat	0.06	-0.09	-0.12	-0.1	-0.19	0.08	0.12	-0.12	**0.75**	
Poultry	0.17	-0.05	-0.15	-0.21	-0.28	0.18	-0.03	**0.62**	**-0.38**	
Innards	0.06	-0.07	-0.14	0.16	0.10	-0.17	0	0	0.01	
Freshwater fish	-0.03	0.12	**0.43**	0.20	0.03	**0.44**	-0.11	0.12	0.16	
Marine fish	-0.05	-0.13	-0.23	-0.05	0.05	**-0.52**	-0.14	0.19	0.08	
Crustacean	-0.02	0.03	-0.05	-0.12	-0.12	-0.03	0.20	**-0.62**	**-0.46**	
Soymilk	-0.08	0.13	0.04	-0.03	-0.07	-0.17	-0.03	-0.11	-0.02	
Tofu	0.29	-0.11	**-0.31**	0.27	**0.38**	**0.31**	0.03	-0.21	-0.04	
Eggs	-0.26	0.02	-0.18	0.11	-0.13	0.02	0.19	0.01	0.03	
Nuts	0.03	-0.27	0.12	-0.13	0.01	-0.07	-0.01	0.02	0	
Carbonated drinks	0.12	-0.21	0.18	**0.45**	-0.29	-0.18	-0.10	-0.07	-0.06	
Pure juices	0.22	-0.05	0.03	-0.03	-0.11	0.10	-0.13	-0.02	-0.01	
Other sugar drinks	-0.15	0.19	-0.16	**-0.49**	**0.38**	0.14	0.06	0.04	0.05	
Candies and chocolates	-0.11	0.13	0.13	0.13	-0.01	0.04	0	0.04	0.04	
Fried dough foods	-0.19	-0.02	0.05	0.03	-0.20	-0.04	0.11	0.04	-0.01	
Pickles	0.22	0.05	-0.03	-0.06	-0.10	-0.09	-0.02	-0.01	0.03	
Processed meat	**-0.36**	0.07	0.06	0.15	0.08	0.25	0.08	0.05	-0.14	
Pastries	0.14	0.23	-0.14	-0.06	-0.04	-0.09	0.02	-0.02	0.07	
Females										
	PC1	PC2	PC3	PC4	PC5	PC6	PC7	PC8	PC9	PC10
Rice	-0.01	-0.03	0	-0.02	-0.08	-0.10	0.15	**0.78**	-0.24	**0.42**
Wheat	0.15	-0.04	0.05	-0.10	-0.12	0	0.22	0.26	-0.07	**-0.64**
Whole grain and mixed beans	0.19	-0.03	-0.01	**-0.42**	-0.09	-0.01	0.23	0.13	0.16	0.01

Potatoes	0.21	0.01	-0.17	**-0.34**	-0.07	0.13	0.21	0.05	0.18	0.04
Fresh vegetables	0.17	**0.40**	0.08	0.25	-0.29	-0.18	0.17	-0.11	-0.02	0.06
Dark vegetables	0.19	**0.35**	0.02	0.24	**-0.32**	-0.20	0.09	-0.09	-0.03	0.16
Mushrooms	0.29	0.15	-0.12	-0.09	-0.16	0.02	0.01	0.01	0.04	-0.05
Fruits	0.16	-0.04	**0.32**	-0.09	-0.04	-0.21	-0.23	0.07	0.06	-0.12
Dairy	0.10	-0.12	**0.30**	-0.18	**-0.31**	-0.09	-0.20	-0.18	0.03	0.17
Yogurt	0.13	-0.13	**0.33**	-0.19	-0.06	-0.11	-0.13	-0.03	0	0.01
Pork	0.22	0.13	-0.07	**0.32**	0.04	-0.06	-0.16	0.11	0.23	-0.16
Meat from other livestock meat	0.28	-0.04	0	0.15	0.16	0.09	**-0.30**	0.19	0.20	-0.04
Poultry	0.26	-0.02	-0.05	0.11	0.03	0.10	**-0.36**	0.29	0.17	-0.04
Innards	0.19	-0.1	**-0.45**	-0.11	-0.23	0	-0.05	-0.05	0.14	0.12
Freshwater fish	0.22	0.25	0.11	0.02	0.13	0	0.13	-0.02	-0.22	0.06
Marine fish	0.26	0.06	0.09	-0.02	0.26	**0.32**	0.04	-0.08	-0.17	0.25
Crustacean	0.26	0.05	0.09	-0.04	**0.36**	0.21	0.17	-0.13	-0.07	0.16
Soymilk	0.17	-0.16	0.15	-0.1	-0.2	0.14	-0.15	-0.03	**-0.39**	-0.13
Tofu	0.27	0.14	0.01	0	0.07	0.14	0.04	-0.04	-0.29	-0.08
Eggs	0.21	0.07	0.11	-0.08	0.17	-0.10	0.02	-0.07	0.15	-0.14
Nuts	0.11	-0.06	0.06	-0.1	**0.34**	**-0.31**	0.12	-0.03	**0.32**	0.12
Carbonated drinks	0.10	-0.29	0.09	**0.34**	-0.02	**0.33**	**0.32**	-0.05	0.18	-0.03
Pure juices	0.13	-0.27	0.17	-0.04	-0.23	0.12	-0.06	-0.12	0.13	0.29
Other sugar drinks	0.08	**-0.36**	0.07	**0.33**	-0.22	0.12	**0.30**	0.04	0.06	-0.07
Candies and chocolates	0.12	-0.29	0.05	0.27	0.08	-0.28	0.02	-0.01	-0.01	0.20
Fried dough foods	0.14	-0.23	-0.21	-0.04	-0.06	-0.09	0.04	-0.19	-0.27	-0.06
Pickles	0.16	-0.10	**-0.48**	-0.03	-0.02	-0.13	-0.03	-0.14	0	0.07
Processed meat	0.15	-0.22	-0.22	0.11	0.15	-0.17	-0.24	-0.03	**-0.39**	-0.05
Pastries	0.10	-0.19	0.07	-0.01	0.19	**-0.50**	**0.30**	-0.03	-0.09	-0.09

	PC11	PC12	PC13	PC14	PC15	PC16	PC17	PC18	PC19	PC20
Rice	-0.12	-0.05	-0.04	-0.18	0.11	-0.14	0.13	-0.04	0.09	0
Wheat	-0.14	0.16	-0.16	-0.16	**-0.37**	-0.1	-0.01	0.20	-0.19	0.14
Whole grain and mixed beans	0.12	0.16	0.2	0.24	0.06	0.18	0.07	-0.24	**-0.36**	-0.28
Potatoes	0.11	-0.17	0.12	0.17	0.23	0.08	-0.08	0.26	-0.05	-0.03
Fresh vegetables	0.06	0.18	0.06	0	0	-0.05	0.03	0.01	-0.04	0
Dark vegetables	0.11	0.21	-0.08	0.06	-0.02	-0.08	0.08	-0.17	-0.11	0.05
Mushrooms	0	-0.18	-0.13	0.01	-0.04	-0.06	-0.14	-0.04	0.13	-0.13
Fruits	**0.39**	-0.07	-0.01	0.11	-0.06	0.04	**0.58**	0.20	**0.32**	-0.09
Dairy	**-0.39**	-0.08	0.19	-0.18	0.15	-0.16	0.10	**0.38**	**-0.32**	0.19
Yogurt	**0.42**	-0.09	0.01	**-0.36**	0.07	-0.29	**-0.50**	-0.12	0.10	-0.13
Pork	-0.25	-0.18	0.23	-0.04	0.24	0.05	-0.12	0.04	0.08	-0.21
Meat from other livestock meat	0.08	0.20	0.1	-0.03	0.02	-0.02	0	-0.06	-0.21	0.19
Poultry	0.02	0.12	0.16	0.06	-0.13	0.17	-0.11	-0.05	0.08	0.20
Innards	0.04	-0.02	-0.12	-0.1	-0.14	-0.02	-0.05	-0.06	0.20	0.12
Freshwater fish	-0.14	-0.17	0.13	-0.06	-0.09	**0.43**	-0.15	**0.35**	0.20	-0.18
Marine fish	0.05	0.05	-0.05	-0.03	-0.23	0	0.03	0.07	-0.01	0.14
Crustacean	0.10	0	0.08	-0.1	-0.09	-0.14	0.23	-0.05	-0.09	0.07
Soymilk	-0.10	0.07	-0.27	**0.37**	**0.42**	0.20	-0.03	-0.16	0.16	0.24
Tofu	-0.02	-0.02	-0.15	0.05	0.12	-0.24	-0.20	0	-0.01	0.04
Eggs	**-0.33**	**-0.40**	-0.27	-0.19	0.06	-0.01	0.28	**-0.49**	-0.06	-0.08
Nuts	-0.18	**0.49**	**-0.44**	-0.01	0.21	-0.01	-0.10	0.24	0.18	-0.08
Carbonated drinks	0.01	0.09	0.18	0.01	**0.32**	-0.24	0.12	-0.01	0.08	0.01
Pure juices	**-0.34**	0.11	0	0.16	**-0.47**	0	-0.07	-0.16	0.20	-0.18
Other sugar drinks	0.11	-0.16	-0.19	-0.03	-0.07	0.08	0.02	0.13	0.07	-0.20
Candies and chocolates	0.19	-0.24	-0.26	0.05	-0.05	**0.35**	-0.14	0.03	**-0.47**	0.14

Fried dough foods	0.01	**0.30**	0.22	**-0.56**	0.14	**0.37**	0.14	-0.15	0.09	-0.04
Pickles	0.06	-0.20	-0.09	0	0	-0.23	0.18	0.21	0.06	0.21
Processed meat	-0.06	0.11	0.07	0.22	-0.04	-0.29	0.06	0.08	-0.2	**-0.55**
Pastries	-0.09	-0.09	**0.42**	0.27	-0.12	-0.11	-0.14	-0.16	0.23	**0.35**

	PC21	PC22	PC23	PC24	PC25	PC26	PC27	PC28	PC29
Rice	0.07	-0.01	0.02	-0.01	0	-0.02	0.01	-0.01	0.03
Wheat	0.19	0.07	0.07	-0.15	-0.05	0.04	-0.04	0.04	-0.07
Whole grain and mixed beans	-0.11	0.16	**0.30**	0.29	-0.04	0.16	-0.02	0	0.11
Potatoes	0.21	-0.08	**-0.57**	-0.28	-0.04	-0.09	0.03	-0.08	-0.11
Fresh vegetables	0.09	0.07	-0.17	-0.05	0	-0.18	-0.25	0.18	**0.61**
Dark vegetables	-0.05	0.11	-0.06	-0.07	-0.03	0.12	0.16	-0.15	**-0.62**
Mushrooms	-0.13	**-0.36**	**0.30**	-0.09	0.12	**-0.30**	**0.55**	0.27	0.07
Fruits	0.08	-0.18	0.03	0	0	0.18	0	-0.04	0.03
Dairy	-0.25	-0.07	0.05	0.04	-0.02	0.01	0.01	0.04	-0.02
Yogurt	0.08	0.27	0.02	-0.06	-0.01	-0.01	-0.08	0.02	-0.04
Pork	0.24	0	0.21	0.01	**-0.54**	0.11	-0.01	-0.13	-0.01
Meat from other livestock meat	-0.01	0	0.02	-0.03	**0.34**	-0.29	0.10	**-0.57**	0.15
Poultry	-0.22	0.11	-0.28	0.20	0.02	0.05	-0.09	**0.55**	-0.15
Innards	-0.27	-0.18	0.21	-0.29	-0.03	0.20	**-0.49**	-0.16	0.04
Freshwater fish	-0.06	0.23	0.18	0	**0.42**	0.02	-0.18	-0.08	-0.14
Marine fish	-0.17	0.23	-0.03	-0.24	**-0.33**	**0.35**	**0.35**	-0.06	0.27
Crustacean	0.08	-0.16	0.17	0.06	-0.24	**-0.48**	**-0.33**	0.14	-0.25
Soymilk	0.06	0.18	0.13	-0.12	-0.12	-0.18	-0.09	-0.01	0.01
Tofu	0.05	**-0.43**	-0.17	**0.52**	0.09	**0.35**	-0.1	-0.14	0.05
Eggs	-0.09	0.16	-0.24	-0.09	0.16	0.07	-0.06	0.03	0.04
Nuts	-0.07	0.01	-0.01	0.04	-0.04	-0.02	0.02	0.06	0

Carbonated drinks	0.09	0.02	0.17	-0.2	**0.35**	0.26	0.01	0.22	-0.03
Pure juices	**0.43**	-0.04	-0.08	0.08	0.05	-0.04	0.02	-0.08	-0.01
Other sugar drinks	**-0.44**	0.1	-0.19	0.28	-0.19	-0.24	0.02	-0.2	0.01
Candies and chocolates	0.23	-0.19	0.08	-0.09	0.02	0.12	-0.02	0.15	0.03
Fried dough foods	0.1	-0.13	-0.14	0.07	-0.03	0.01	0.18	0.01	0.01
Pickles	0.27	**0.47**	0.11	**0.36**	0.07	-0.04	0.12	0.05	0.04
Processed meat	-0.13	0.05	-0.12	-0.23	0.05	-0.03	-0.06	0.08	-0.02
Pastries	-0.15	-0.06	-0.04	-0.03	-0.04	-0.02	0.07	-0.08	0.03
